# Localization of *Arabidopsis* Glucan Synthase-Like 5, 8, and 12 to plasmodesmata and the GSL8-dependent role of PDLP5 in regulating plasmodesmal permeability

**DOI:** 10.1080/15592324.2022.2164670

**Published:** 2023-01-16

**Authors:** Behnaz Saatian, Susanne E. Kohalmi, Yuhai Cui

**Affiliations:** a Agriculture and Agri-Food Canada, London Research and Development Centre, London, Ontario, Canada; b Department of Biology, Western University, London, Ontario, Canada

**Keywords:** plasmodesmata, glucan synthase-like, callose, symplastic trafficking, plasmodesmal permeability

## Abstract

Cell-to-cell communication via membranous channels called plasmodesmata (PD) plays critical roles during plant development and in response to biotic and abiotic stresses. Several enzymes and receptor-like proteins (RLPs), including *Arabidopsis thaliana* glucan synthase-likes (GSLs), also known as callose synthases (CALSs), and PD-located proteins (PDLPs), have been implicated in plasmodesmal permeability regulation and intercellular communication. Localization of PDLPs to punctate structures at the cell periphery and their receptor-like identity have raised the hypothesis that PDLPs are involved in the regulation of symplastic trafficking during plant development and in response to endogenous and exogenous signals. Indeed, it was shown that PDLP5 could limit plasmodesmal permeability through inducing an increase in callose accumulation at PD. However, mechanistically, how this is achieved remains to be elucidated. To address this key issue in understanding the regulation of PD, physical and functional interactions between PDLPs and GSLs (using the PDLP5–GSL8/CALS10 pair as a model) were investigated. Our results show that GSL8/CALS10 plays essential roles and is required for the function and plasmodesmal localization of PDLP5. Furthermore, it was demonstrated that the localization of PDLP5 to PD and its function in inducing callose deposition are GSL8-dependent. Importantly, our transgenic study shows that three key members of the GSL family, i.e., GSL5/CALS12, GSL8/CALS10, and GSL12/CALS3, localize to PD and co-localize with PDLP5, suggesting that GSL8/CALS10 might not be the only callose synthase with the determining role in PD regulation. These findings, together with our previous observation showing the direct interaction of GSL8/CALS10 with PDLP5, indicate the pivotal role of the GSL8/CALS10-PDLP5 interplay in regulating PD permeability. Future work is needed to investigate whether the PDLP5 functionality and localization are also disrupted in *gsl5* and *gsl12*, or it is just *gsl8*-specific.

## Introduction

Different from animal cells, plant cells have stiff cell walls and do not move after cell division. Therefore, the determination of a plant cell’s identity largely relies on positional cues. Cell-to-cell communication and exchange of information between cells are required for the survival and growth of plants. Plant cells are interconnected via narrow channels of plasmodesmata (PD) that facilitate symplastic movement of nutrients and signaling molecules.^1^ PD pores that are formed between cells, allowing macromolecular trafficking through the cell wall barrier, are more than fixed passive channels. Instead, they dynamically regulate symplastic connectivity through allowing passage of molecules with different sizes.^2^ PD are regulated by the production and/or degradation of callose.^3,4^ Genetic, molecular, and biochemical studies during the last decades have identified a number of important factors and implicated the key role of callose equilibration at PD in the regulation of their permeability and symplastic movement. Nevertheless, how the identified players are physically and functionally linked to perceive and transduce the endogenous signals and subsequently regulate the callose homeostasis is not well-understood.

PD-located protein 5 (PDLP5) in *Arabidopsis* belongs to a small subfamily of receptor-like proteins (RLPs) with a distinct N-terminal domain exposed to the apoplast, a transmembrane domain in the middle, and an extremely short C-terminal cytoplasmic domain. The PDLP family in *Arabidopsis* has eight members, all of which are targeted to PD. PDLP5 interaction with other callose modifying enzymes can potentially initiate signal transduction.^5^ Glucan synthase-likes (GSLs), also known as callose synthases (CALSs), are enzymes responsible for synthesizing callose in response to different developmental, physiological, and environmental signals and in various tissues in plants.^6^ Out of the 12 GSLs in *Arabidopsis*, GSL4/CALS5, GSL6/CALS1, GSL7/CALS7, GSL8/CALS10, and GSL12/CALS3 have so far been implicated in the regulation of PD.^3,7,8^ It should be noted that although all the *Arabidopsis* callose synthases mentioned above are required for the regulation of PD permeability, these enzymes are likely to function in discrete signaling pathways. Despite GSLs’ critical role in plant development, characterizing their subcellular localization using stable transgenic plants has remained a challenge.

It is well documented that PDLP5 negatively regulates PD size exclusion limit (SEL) by inducing callose deposition.^7,9–11^ Wang et al. showed the critical role of PDLP5 in callose accumulation at PD in response to salicylic acid (SA) and during the plant response to microbial pathogens.^11^ More recently, Cui and Lee provided the first direct evidence on the requirement of PDLP5 for GSL4/CALS5 function in maintaining the basal plasmodesmal permeability but not for reactive oxygen species (ROS)-induced PD regulation. On the contrary, PDLP5 is required for GSL6 function at PD in response to SA.^7^ Yet, how PDLP5 affects the activities of GSLs is not clear. We have recently demonstrated that GSL8 and PDLP5 interact *in vivo*.^12^ Based on the findings, we hypothesized that PDLP5 might relay cellular signals to GSL8/CALS10, through physical association, to induce callose deposition and regulate PD size exclusion limit. Here we provide evidence, for the first time, implicating a strategy employed by the plasmodesmal regulator PDLP5 to induce callose accumulation at PD through the callose biosynthesis enzyme GSL8/CALS10.

## Results

### GSL5/CALS12, GSL8/CALS10, and GSL12/CALS3 co-localize with PDLP5 at PD

**Figure 1.**
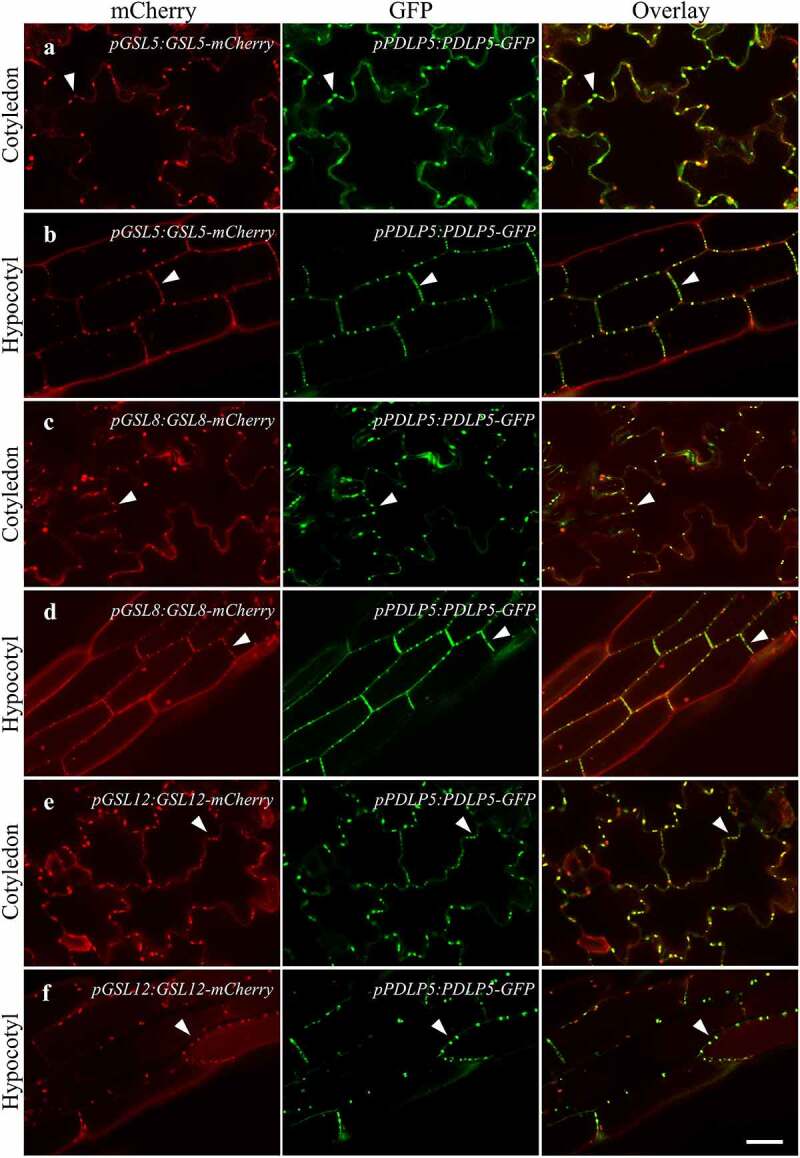
Subcellular localization of GSL5, GSL8, and GSL12 and their co-localization with PDLP5 at PD. Arabidopsis double transgenic plants expressing mCherry-tagged GSLs and GFP-tagged PDLP5 were examined under confocal microscope: GSL5 (a-b), GSL8 (c-d), and GSL12 (e-f). Cotyledons and hypocotyls of 2-week-old seedlings were used. Colocalization of PDLP5 with GSL5, GSL8 or GSL12 are indicated with white arrowheads. Scale bars = 40 µm.

To gain insight into the GSLs’ localization along the plasma membrane and the mechanism they use to regulate the level of callose at PD, we investigated the subcellular localization of GSL5/CALS12, GSL8/CALS10, and GSL12/CALS3. These GSLs belong to three distinct subfamilies in the *Arabidopsis* GSL family phylogenetic tree.^12^ GSL5/CALS12 is the best characterized member of the family and is known as the callose synthase required for callose formation in response to fungal pathogens.^13–15^ The *GSL5* gene only has three exons, which is different from most members of the GSL family.^16^ GSL8/CALS10 and GSL12/CALS3 both have been implicated in determining PD size exclusion limit and controlling molecular trafficking.^3,8,12^ Double stable transgenic plants (*pPDLP5:PDLP5-GFP pGSL5:GSL5-mCherry, pPDLP5:PDLP5-GFP pGSL8:GSL8-mCherry*, and *pPDLP5:PDLP5-GFP pGSL12:GSL12-mCherry*) were generated (see Materials and Methods for details) and examined under confocal microscope after being confirmed by genotyping. All the three mCherry-tagged GSLs showed punctate pattern at the cell periphery in the cotyledon and hypocotyl of 2-week-old seedlings (Figure 1). These spots co-localize with GFP-tagged PDLP5, which was used as a PD marker (Figure 1). These data indicate that GSL5/CALS12, GSL8/CALS10, and GSL12/CALS3 are localized to PD. Of note, we confirmed that the GSL8–mCherry fusion is functional *in vivo*, as evidenced by the fact that it could complement a *gsl8*/*cals10* loss-of-function mutant (*essp8*) (Supplementary Figure S1).

## Callose induction by PDLP5 at PD is partially GSL8-dependent

**Figure 2.**
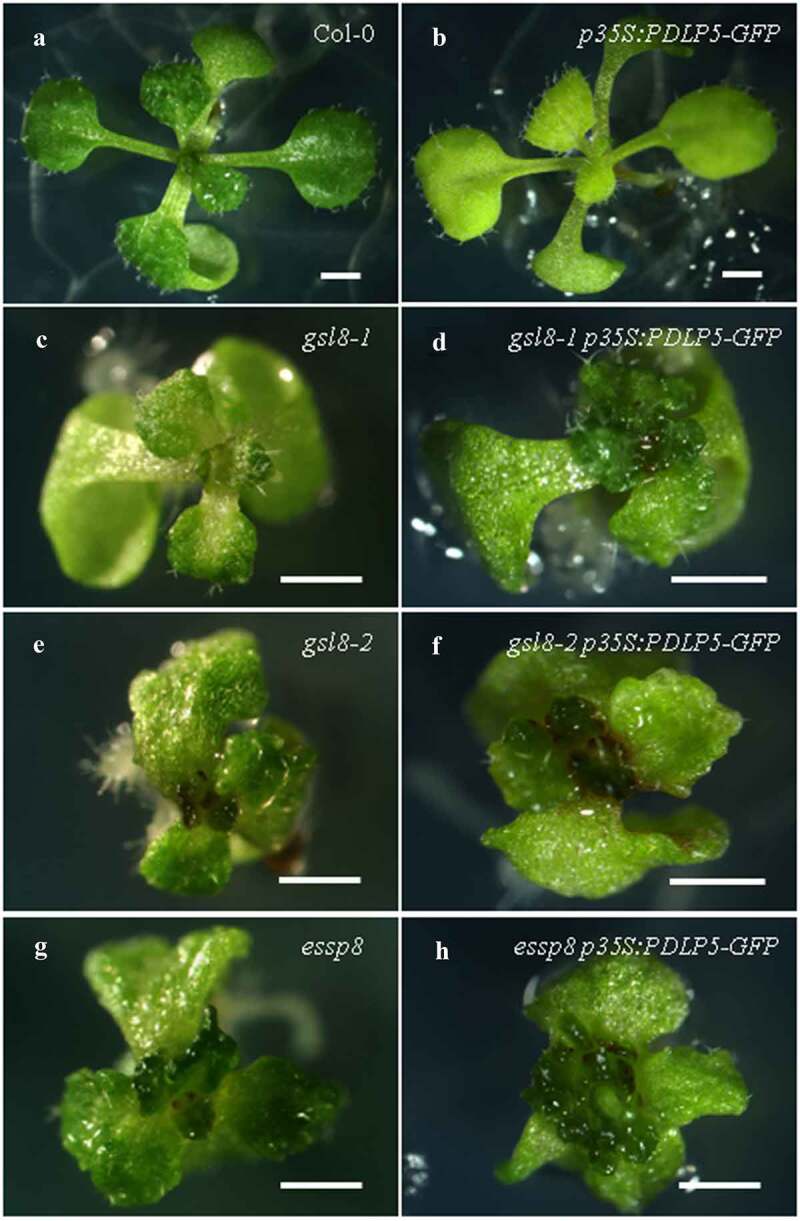
The morphological phenotype of *PDLP5 overexpression* lines (*PDLP5OE*). (a-b) *PDLP5OE* seedlings are yellowish (b) compared to WT Col-0 (a). (c-h) Overexpression of *PDLP5* in the *gsl8-1* (d), *gsl8-2* (f) and *essp8* (h) backgrounds did not cause any noticeable morphological changes compared to the mutant alone: *gsl8-1* (c), *gsl8-2* (e), and *essp8* (g). Scale bar = 1 cm.

**Figure 3.**
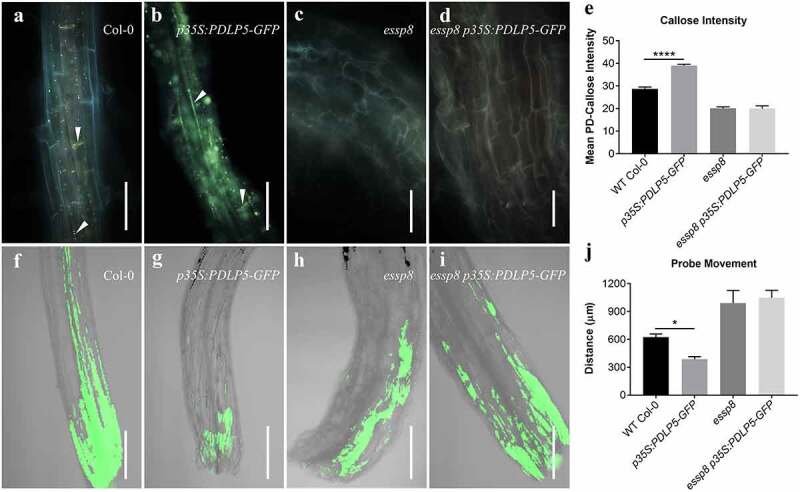
The overexpression of *PDLP5* induces callose deposition at PD and restricts cell-to-cell movement. (a-b) Aniline blue staining for callose showing that *PDLP5* overexpression induces callose accumulation at PD in the primary root of 5-day-old seedlings (b) relative to WT Col-0 (a). Callose depositions are indicated with white arrowheads. (c-d) The overexpression of *PDLP5* in *essp8* seedlings caused no increase in callose deposition (d) compared to *essp8* (c). White arrowheads and arrows indicate callose deposition at PD and the cell plate, respectively. (e) Quantification of the PD callose levels is shown in a-d, confirming the significant increase in *p35S:PDLP5-GFP* seedlings relative to WT Col-0, and no difference between *essp8 p35SPDLP5-GFP* and *essp8*. Values reported are the mean ± SEM (*n* = 10). The asterisks denote significant difference (one-way ANOVA, ****P < .0001). (f-i) SEL assay using the fluorescein probe showing the restricted movement of the probe in *PDLP5OE* hypocotyls (g) relative to WT Col-0 (f). Movement of the probe did not show any difference in the *essp8 PDLP5OE* hypocotyl (i) compared to *essp8* (h). (j) Quantification of the probe movement, confirming its significant decrease in *PDLP5OE* relative to WT Col-0. Values reported are the mean ± SEM (*n* = 5). The asterisks denote significant difference (one-way ANOVA, *P < .05). Scale bars = 500 µm (a-b), 50 µm (c-d), 400 µm (f-j).

Given the colocalization of PDLP5 and GSL8/CALS10 at PD and their physical interaction shown previously,^12^ we next examined their functional/genetic relationship. It has been shown previously that *PDLP5* overexpression (*PDLP5OE*) leads to higher accumulation of callose at PD, with the concomitant reduction of cell-to-cell movement.^9,10^ Interestingly, we have also recently observed a significant decrease in callose deposition at PD in the primary roots of a *gsl8*/*cals10* loss-of-function mutant (*essp8*).^12^ Based on these previous findings, we investigated whether *PDLP5* overexpression in *gsl8* background can, at least partially, restore callose deposition at PD, and thus the size exclusion limit, and rescue the severe phenotype of *gsl8*/*cals10* seedlings. *PDLP5* overexpression in wild-type (WT) background led to yellowish color formation throughout the seedling phase (Figure 2a-b), consistent with a published observation;^9^ but it could not rescue the *gsl8*/*cals10* phenotype (Figure 2c-h). Next, we examined the effect of *PDLP5* overexpression on callose accumulation at PD by aniline blue staining of the primary roots. Overexpressing *PDLP5* in the WT background led to a significant increase in basal callose (Figure 3a-b and e); however, no change was observed in the *essp8* mutant background (Figure 3c-e). Similarly, using a previously described SEL assay for estimating plasmodesmal permeability by measuring the cell-to-cell movement of a fluorescence dye,^12,17^ we saw that the movement of the dye was less extensive in the hypocotyl of *PDLP5OE* seedlings than that in WT Col-0 (figure 3f-g and j), indicating an increase in callose at PD induced by PDLP5 overexpression. In contrast, in the *essp8 PDLP5OE* hypocotyls, there was no significant change compared to that in the *essp8* null mutants (Figure 3h-j), suggesting no new callose deposition. Together, these findings indicate that callose induction by PDLP5 at PD is likely through GSL8/CALS10.

## PDLP5-GFP is partially mislocalized in *gsl8*/*cals10* seedlings

**Figure 4.**
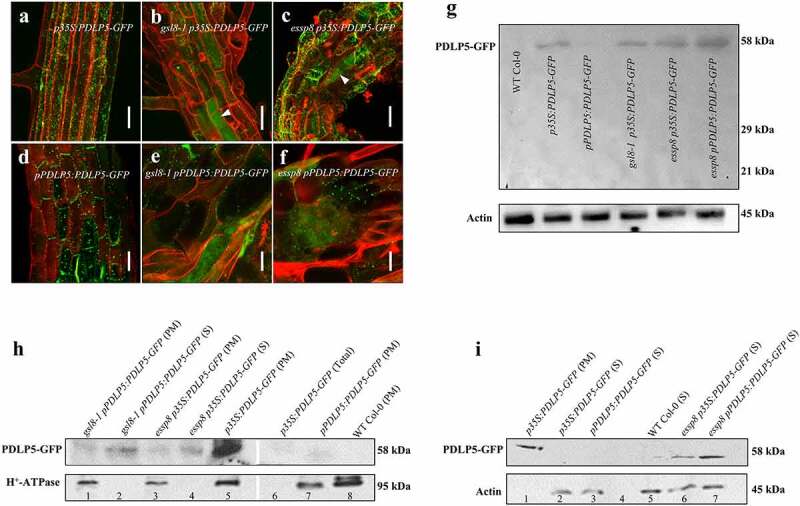
The mislocalization of PDLP5-GFP in *gsl8* seedlings. (a-f) PDLP5-GFP was localized at PD, identified as punctate patterns at cell periphery in *PDLP5OE* primary roots (a), whereas in *gal8-1* (b) and *essp8* (c) backgrounds, PDLP5-GFP signal was detected both at PD and in the cytoplasm. The generation of PDLP5-GFP tagged lines under the control of its native promoter showed the same pattern with localization to PD in hypocotyls of the *pPDLP5:PDLP5-GFP* plants in WT Col-0 (d), and localization to both PD and the cytoplasm in the *gsl8-1* (e) and *essp8* (f) hypocotyls. (g) Immunoblots showing that PDLP5-GFP is intact in both overexpression and native promoter-driven lines. (h-i) Western blot showing the detection of PDLP5-GFP signals form different cellular fractions. Note: PM fraction was detected in both WT Col-0 (lanes 5 and 7) and *gsl8* backgrounds (lanes 1 and 3) (h). The cytosolic fractions in lanes 2 and 4 are used as the negative control for the PM fraction. The total protein from the PDLP5-GFP overexpression line (lane 6) is used as a positive control where WT Col-0 is included as a negative control (lane 8). The cytosolic (s) fraction PDLP5-GFP signal was only detected in the *gsl8* background (H: lanes 2 and 4, I: lanes 6 and 7). No PDLP5-GFP signal is detected in the WT background (I: lanes 2 and 3). The PM fraction from the PDLP5_GFP overexpression line (I: lane 1) is used as a negative control for the cytosolic fraction where WT-Col-0 is included as a negative control (I: lane 5). Actin and H^+^-ATPase were used as loading controls for total and cytosolic and plasma membrane fractions, respectively. All imaging and Western blots analysis were repeated at least three times to confirm. Scale bars = 50 µm.

We have previously shown the subcellular localization of PDLP5-GFP at PD when it is overexpressed (*p35S:PDLP5-GFP*) in WT and *gsl8* mutant backgrounds,^12^ where PDLP5-GFP fluorescent signals were detected as punctate dots along the cell periphery of primary roots. Later, we further examined PDLP5-GFP subcellular localization and noticed that the PDLP5-GFP signal in some primary root cells of the *gsl8 PDLP5OE* seedlings was localized both at the cellular boundaries, as well as in cytoplasm (Figure 4a-c), suggesting that PDLP5 localization might be disrupted in *gsl8* null mutants. To obtain a more accurate view on localization of PDLP5-GFP within the cells of the primary roots in WT and *essp8* seedlings, Z-stack assemblies of 1-µm slices covering the primary root were used for the three-dimensional visualization. A detailed analysis of PDLP5-GFP localization confirmed our earlier observation in two-dimensional images, as the signals were detected only at PD in the WT (Supplementary Movie S1); however, in *gsl8* null mutant seedlings, PDLP5-GFP signals were detected both at PD and in the cytoplasm (Supplementary Movies S2-S3). In order to eliminate the possibility of *PDLP5* overexpression leading to its mislocalization to cytoplasm, we generated PDLP5-GFP tagged lines under the control of its native promoter (*pPDLP5:PDLP5-GFP*) in WT Col-0, *gsl8-1*, and *essp8* genetic backgrounds, respectively. Similarly, PDLP5-GFP signals in *gsl8* seedlings were detected both at the cell periphery, associated with PD, and in the cytoplasm (Figure 4d-f). The analysis of the three-dimensional images further confirmed the localization of PDLP5-GFP at the cell membrane in the WT (Supplementary Movie S4), with its partial mislocalization in *gsl8* mutant seedlings (Supplementary Movies S5-S6).

To rule out the possibility that the cytoplasmic GFP signal might be obtained from the cleavage product of PDLP5-GFP, a Western blot was performed, and it was revealed that the PDLP5-GFP fusion protein was intact in both transgenic lines (*p35S:PDLP5-GFP* and *pPDLP5:PDLP5-GFP*) (Figure 4g). Next, we performed Western blots on different cellular fractions including membrane proteins and cytosolic proteins to confirm that PDLP5-GFP is indeed localized into the cytoplasm in *gsl8*/*cals10* seedlings. As shown in Figure 4h-i, PDLP5-GFP was detected in both the cytosolic and the membrane fractions in *gsl8*/*cals10* seedlings, while in the WT Col-0, the signal was only detected in the membrane fraction. These data provide further support for the mislocalization of PDLP5 in *gsl8*/*cals10* seedlings.

## Discussion

Multicellular plants’ life and survival rely on direct cell-to-cell communication between neighboring cells mediated by PD. The regulatory mechanisms underlying the size control of PD are, however, not well-understood yet. Callose levels at the “neck” regions of PD modulate plasmodesmal permeability. Recent studies have identified and characterized callose synthases that are involved in regulating plasmodesmal permeability,^3,7,17^ and a direct interaction of PDLP5 and GSL8/CALS10 has been implicated in our earlier work.^12^ It was suggested that PDLP5 is likely to require specific callose synthase(s) to modulate the level of plasmodesmal callose,^7^ but the specific GSLs have never been identified, nor have their roles demonstrated.

**Figure 5.**
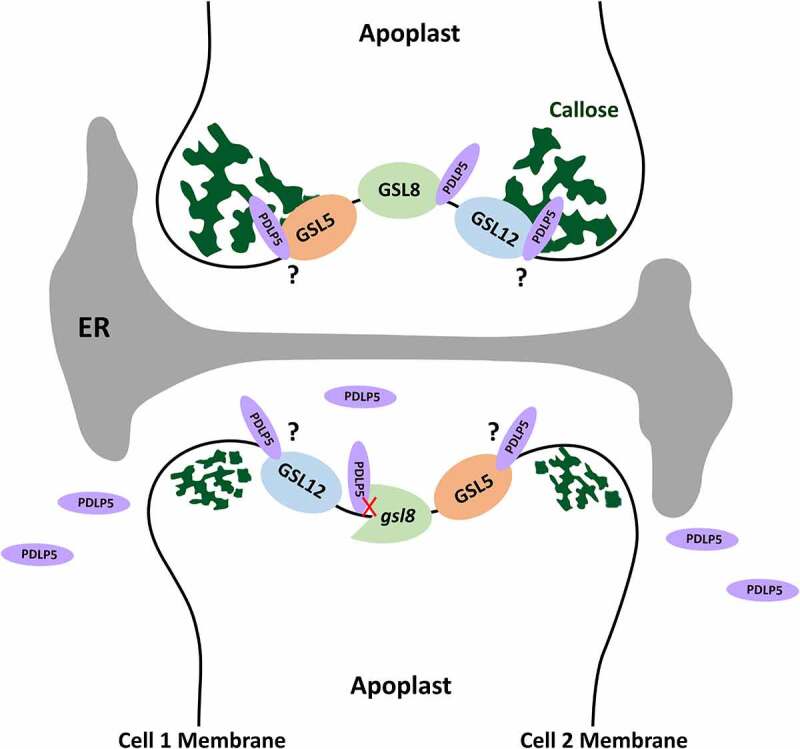
Proposed model of PD aperture regulation. PDLP5 regulates both callose deposition at PD and the basal plasmodesmal permeability through forming a functional protein complex with GSL8/CALS10 (top). In gsl8/cals10 seedlings, PDLP5 loses its interaction with GSL8 leading to its partial mislocalization (bottom). Partial PDLP5 mislocalization in gsl8/cals10 seedlings indicates the likelihood of PDLP5 interaction with other GSLs including GSL5/CALS12 and GSL12/CALS3 that needs to be further investigated.

The overexpression of *PDLP5* restricts symplastic trafficking and induces callose accumulation at PD.^5,9,18^ The reduction of PD connectivity associated with *PDLP5* overexpression leads to defects in systematic acquired disease resistance.^19^ Therefore, the precise regulation of PD connectivity and signaling pathways play critical roles in both plant development and defense.^20^ Our current study provides direct evidence that callose deposition at PD and regulation of plasmodesmal permeability by PDLP5, as well as its subcellular localization, are, at least partially, GSL8/CALS10-dependent. *PDLP5* overexpression in WT seedlings induces callose accumulation at PD with concomitant restriction of intercellular movement (Figure 3). In contrast, the overexpression of *PDLP5* in *gsl8*/*cals10* seedlings fails to improve either the callose accumulation at PD or the symplastic trafficking defects (Figure 3). Along these lines of evidence and our previous study,^12^ we speculate that PDLP5 is likely to form a functional protein complex with GSL8/CALS10 to regulate callose deposition at PD and regulate the basal plasmodesmal permeability. The partial mislocalization of PDLP5 in *gsl8*/*cals10* seedlings raises the hypothesis of PDLP5 interaction with other GSLs including GSL5/CALS12 and GSL12/CALS3 and the likelihood of an overlapping function between different GSLs that need to be further explored (Figure 5). Although PDLP5 partially depends on GSL8/CALS10 to localize at PD and execute callose induction at PD, GSL8/CALS10 is also likely to affect basal callose deposition at PD and symplastic trafficking through a PDLP5-independent pathway, as previously shown.^7^ It has been demonstrated that *gsl6* mutations can completely suppress the callose accumulation at PD by PDLP5 overexpression in response to infection and during SA-induced callose production.^7^ It still needs to be investigated whether PDLP5 and GSL6/CALS1 can form a functional protein complex in response to certain environmental stimuli inducing SA biosynthesis. It was also found that GSL4/CALS5 requires PDLP5 for maintaining the basal plasmodesmal permeability. By analyzing *gsl8-1 PDLP5OE, essp8 PDLP5OE, gsl8-1 PDLP5*, and *essp8 PDLP5* stable transgenic lines, we have shown that GSL8/CALS10 is required for regulating basal plasmodesmatal permeability. Additionally, PDLP5 partially relies on a functional GSL8/CALS10 to localize into PD and induces callose accumulation.

Here, we also reported the successful generation of stable transgenic lines expressing mCherry-tagged GSLs (GSL5/CALS12, GSL8/CALS10, and GSL12/CALS3) under their respective native promoters and demonstrated their localization at PD. Associations of GSL8/CALS10 and GSL12/CALS3 with PD regulation have been previously implicated,^3,8,12^ but their plasmodesmal localization under the control of their native promoters have never been experimentally demonstrated. Vatén et al.^3^ previously showed the localization of GFP-GSL12 at plasmodesmata where the transgene was under the control of a constitutive promoter, not its native promoter. GSL5 is known as a putative regulator of PD callose accumulation in response to SA and pathogens;^21,22^ however, there is no direct evidence suggesting its connection with basal callose deposition at PD. To our knowledge, this is the first report on the subcellular localization of GSLs through stable transgenic plants with transgenes under the control of their native promoters. Such a task was considered to be technically challenging, given the large size of these genes.^7^ These materials, both the constructs and the transgenic seeds, will be useful genetic resources for the plant biology community.

GSLs play critical roles in plant development by modulating callose deposition at PD and controlling the symplastic movement of developmental signals and different molecules and nutrients. Our study, for the first time, implicates a strategy employed by the plasmodesmal regulator PDLP5 to induce callose accumulation at PD through the callose biosynthesis enzyme GSL8/CALS10. It is important in the future to investigate whether the PDLP5 functionality and localization are disrupted in *gsl5* and *gsl12* mutants as well, or whether it is *gsl8*-specific. Studying different combinations of PD-related GSLs with PDLP5 can reveal more details on how cell-to-cell communication is regulated.

## Materials and methods

### Plant materials and growth conditions

*Arabidopsis thaliana* plants were grown at 22°C under a 16 h/8 h light/dark cycle. The *gsl8* mutant lines were all propagated as heterozygotes due to their homozygous lethality.^8,12,23–26^ Seeds for WT Col-0 and T-DNA lines used in this study including *gsl8-1* (SALK-111500) and *gsl8-2* (SALK-109342) were obtained from the Arabidopsis Biological Resource Center, as described previously.^12^

## Generation of transgenic plants

The PDLP5-GFP transgene constructs were generated using the Gateway™ system (Invitrogen).^27^ The generation of *p35S:PDLP-GFP* has been described previously.^12^ To create the *pPDLP5:PDLP5-GFP* translational fusion construct, a 2.3-kb genomic fragment harboring the native promoter and the 5’ untranslated region (UTR), the genomic sequence of *PDLP5* excluding the STOP codon was amplified from *Arabidopsis* gDNA and cloned into the pMDC107 vector.^27^ The translational fusion cassette for *GSL5, GSL8*, and *GSL12* was generated by synthesizing the 2.5–3-kb sequence upstream of the start codon harboring the native promoter and the 5’ UTR, plus the coding sequence from *GSL5, GSL8*, or *GSL12* without the stop codon and C-terminally fused to *mCherry de novo* (BIO BASICS Int.) (*pGSL5:GSL5-mCherry; pGSL8:GSL8-mCherry*; and *pGSL12:GSL12-mCherry*). The synthesized cassettes were then subcloned into a modified yeast-compatible expression vector by homologous recombination.^28^ Stable transgenic *Arabidopsis* plants were produced using these transgene constructs. Double stable transgenic plants expressing both PDLP5 and GFP/GSLs-mCherry were generated by crossing the *pPDLP5:PDLP5-GFP* with the *pGSL5:GSL5-mCherry, pGSL8:GSL8-mCherry*, or *pGSL12:GSL12-mCherry* transgenic plants. The translational cassettes for the generation of all transgenic *Arabidopsis* plants were obtained using the floral dipping method.^29^ Due to the lethality of the homozygous *gsl8* mutant lines, heterozygous plants, confirmed by genotyping, were used for the generation of transgenic plants. A list of all the primers used for genotyping and cloning is provided in Supplementary Table S1.

## Histochemical assays

Callose staining was carried out as previously described.^12^ Briefly, a stock solution of 0.1 mg/ml aniline blue fluorochrome (Biosupplies Australia PTY Ltd.) was prepared in distilled water. Prior to use, the stock solution was diluted 1:3 with 0.1 M K_3_PO_4_, pH 12.0. Roots of 5-day-old *Arabidopsis* seedlings were incubated with the fluorochrome staining solution for 30 min and then washed with 0.1 M K_3_PO_4_, pH 12.0 buffer, and imaged on a Zeiss Axioscope 2 (Zeiss, Germany) compound fluorescence microscope using a UV laser. The microscope was integrated with a Nikon DS-Ri2 digital camera using the ACT-1 software (Nikon, Japan). Five-day-old seedlings were used for callose staining.

The PD size exclusion limit assay has been described previously.^12^ Seeds were allowed to germinate in the dark for 7 d. Dextran, fluorescein, 10,000 MW, Anionic (ThermoFisher Scientific) was dissolved in Tris–EDTA buffer, pH 8.0, at a concentration of 50 mg/ml. Prior to use, the stock was diluted in Tris–EDTA buffer at a ratio of 1:10. The hypocotyls were obtained by cutting the seedlings at the hook. For each sample, 1 μl of the diluted probe was injected into the hypocotyl at the cut site using a Hamilton Gastight syringe (Hamilton). The movement of the probe was analyzed immediately by imaging on a Leica TCS SP2 Laser Scanning confocal microscope (Leica) using 488 nm excitation and 515–530 nm emissions. The distance of probe movement was determined by measuring the distance between the cut site and the furthest fluorescent signal trafficking via symplastic movement.

To stain the cell walls with propidium iodide (PI),^30^ *Arabidopsis* roots were immersed in 1 μg/ml PI solution for 3 min. To visualize both cell walls and nuclei, 100 μg/ml PI solution was used. Roots were stained for at least 5 min and rinsed twice with distilled water. PI-stained roots were imaged on a Leica TCS SP2 Laser Scanning confocal microscope (Leica, Germany) using 543 nm excitation and 610–630 nm emission.

## Protein isolation and Western blot

For isolation of total, soluble, or plasma membrane (PM) proteins, 2-week-old seedlings were homogenized in a buffer containing 150 mM Tris–HCL, pH 8.5, 290 mM sucrose, 25 mM EDTA, 10 mM dithiothreitol (DTT), 1× protease inhibitor cocktail (Roche), 1 mM phenylmethylsulfonyl fluoride (PMSF), and 2% polyvinylpolypyrrolidone (PVPP). Crude lysate was filtered through cheesecloth and then centrifuged at 10,000 *g* for 10 min at 4°C. The obtained clear supernatant is the total protein. Next, total protein fraction was centrifuged at 100,000 *g* for 1 h at 4°C. The supernatant comprises the soluble fraction, and the pellets comprise the microsomal membrane fraction. The pellets were washed with homogenization buffer (without PVPP) to remove any soluble protein contaminations. The PM protein was enriched using an aqueous two-phase partitioning procedure from the total microsomal membrane, as described previously.^31^ The final phase was centrifuged at 100,000 *g* for 1 h at 4°C to precipitate the PM proteins. The pellets were resuspended in PM resuspension buffer (5 mM phosphate buffer, pH 7.8, 1 mM DTT, 1 mM EDTA, 1× protease inhibitor cocktail, and 1 mM PMSF). Finally, the peripheral PM proteins were stripped as described before.^32^ The total, soluble, and PM protein fractions were analyzed using standard sodium dodecyl sulfate polyacrylamide gel electrophoresis (SDS-PAGE, 10% gel) followed by immunoblotting with anti-GFP (Abcam), anti-H^+^-ATPase (PM protein control, Agrisera), and anti-actin (total and soluble protein control, Agrisera) antibodies. The expected molecular weights of the tested proteins are as follows: PDLP5-GFP (58 kDa); Actin (45 kDa); and H^+^-ATPase (95 kDa).

## Microscopy and image processing

To visualize PDLP5-GFP, GSL5-mCherry, GSL8-mCherry, and GSL12-mCherry, the primary roots, hypocotyls, or cotyledons of 5-day-old seedlings were imaged with a Leica TCS SP2 laser scanning confocal microscope (Leica). GFP signals were imaged using 488 nm argon laser excitation and was detected at 500–535 nm. mCherry signals were imaged using 543 nm He/Ne laser excitation and was detected at 560–630 nm. Z-stack confocal images were used to generate 3D and video by the Imaris® software (version 7.6.1, Bitplane AG, Switzerland).

Seedlings’ images were captured by a Nikon SMZ1500 (Nikon) dissecting microscope integrated with a Nikon DS-Ri2 digital camera using the ACT-1 software (Nikon). Imaging for the detection of aniline blue-stained plasmodesmal callose was performed on a Zeiss Axioscope 2 (Zeiss, Germany) compound fluorescence microscope using a UV laser. The microscope was integrated with a Nikon DS-Ri2 digital camera using the ACT-1 software (Nikon). TIFF format at a resolution of 3840 × 3072 pixels was used for capturing all images. The ImageJ software^33^ was used for callose quantification as described previously.^34^ At least three biological replicates were used for all experiments.

## Supplementary Material

Supplemental Material

## Accession numbers

Sequence data used in this article can be found on the Arabidopsis Information Resource (TAIR) database under the following accession numbers: *GSL5*/*CALS12* (AT4G03550), *GSL8*/*CALS10* (AT2G36850), *GSL12*/*CALS3* (AT5G13000), and *PDLP5* (AT1G70690).
